# Exploring Protein Functions of Gut Bacteriome and Mycobiome in Thai Infants Associated with Atopic Dermatitis Through Metaproteomic and Host Interaction Analysis

**DOI:** 10.3390/ijms252413533

**Published:** 2024-12-18

**Authors:** Thanawit Chantanaskul, Preecha Patumcharoenpol, Sittirak Roytrakul, Amornthep Kingkaw, Wanwipa Vongsangnak

**Affiliations:** 1 Genetic Engineering and Bioinformatics Program, Graduate School, Kasetsart University, Bangkok 10900, Thailand; thanawit.chant@ku.th; 2 Department of Zoology, Faculty of Science, Kasetsart University, Bangkok 10900, Thailand; preecha.pa@ku.th; 3 Functional Ingredients and Food Innovation Research Group, National Center for Genetic Engineering and Biotechnology, National Science and Technology Development Agency, 144 Thailand Science Park, Phaholyothin Road, Pathum Thani 12120, Thailand; sittiruk@biotec.or.th; 4 Interdisciplinary Graduate Program in Bioscience, Faculty of Science, Kasetsart University, Bangkok 10900, Thailand; amornthep.ki@ku.th; 5 Omics Center for Agriculture, Bioresources, Food and Health, Kasetsart University (OmiKU), Bangkok 10900, Thailand

**Keywords:** atopic dermatitis, metaproteomics, human gut, microbiome, mycobiome

## Abstract

Atopic dermatitis (AD), a prevalent allergic skin condition in children, has been closely associated with imbalances in the gut microbiome. To investigate these microbial alterations and their functional implications, we investigated protein expression, functions and interactions of the gut bacteriome and mycobiome as well as the human proteome in Thai infants with AD using integrative metaproteomic and host interaction analysis. As we observed, probiotic species, such as *Lactobacillus acidophilus* and *Bacteroides salyersiae*, were reduced in abundance in the AD group while key pathogenic bacteria and fungi, such as *Streptococcus constellatus* and *Penicillium chrysogenum*, increased in abundance. Additionally, the functional analysis of expressed proteins was enriched in response to stress and DNA repair in the bacteriome and ribosome biogenesis-related processes in the mycobiome of the AD group, potentially associated to increased reactive oxygen species (ROS), intestinal inflammation, fungal growth and microbial dysbiosis. Further, a protein–protein interactions (PPIs) network analysis incorporating the human proteome revealed 10 signature proteins related to stress and immune system processes associated with AD. Our findings propose the interactions of the key species and signature protein functions between the gut microbes and the human host in response to AD in Thai infants. To our knowledge, this study serves as the first framework for monitoring bacteriome–mycobiome–human gut studies associated with AD and other allergic diseases in infants.

## 1. Introduction

Atopic dermatitis (AD) is a common allergic disease with a rising incidence. Its symptoms include red rashes, eczematous dermatitis, dry skin and flaky skin, particularly on the face, arms and legs. Although genetic disorders in various positions may contribute to AD, the exact causes remain unclear, similar to other allergies. Chronic itching, infection-related complications and medical expenses significantly impact the quality of life for the patients and their families [1,2]. The microbiome encompasses the genomes of all the microbes in specific environments, including intensely different species of bacteria and fungi [3]. In the human body, the microbiome is found in the cavities and epithelial surfaces in contact with the external environment [4]. Research shows that the microbiome is essential for normal bodily functions which are integral to health. An imbalanced microbiome may play a key role in a number of diseases, particularly metabolic ones [5,6,7]. Despite medical advancements, understanding AD remains challenging, necessitating further studies on the microbiome. One challenge is understanding the functionality of the microbial communities and their impact on the host [8]. Metagenomics has facilitated the study of microbiome compositions using 16S rRNA gene sequencing and ITS2 sequencing while metaproteomics is also a powerful tool for studying protein expression and functions of the microbiome [9].

Our earlier study was based on 16S rRNA gene sequencing of the gut microbiome for healthy and AD infant groups [10]. The study showed *Lachnospiraceae* and *Butyricicoccaceae* to be significantly enriched in the AD group. Moreover, *Ruminococcus*, *Anaerostipes* and *Butyricicoccus*, such as *Anaerostripes hadrus* and *Ruminococcus gnavus,* were also found to be significantly higher in abundance in the AD group with inflammatory diseases [10]. Moreover, a metaproteomic analysis investigating different key proteins playing metabolic functional roles in the microbiome of both infant groups revealed significant differences in protein expression. For instance, triosephosphate isomerase in *Bifidobacteriaceae* in *Alloscardovia* and demethylmenaquinone methyltransferase in Bacteroides were shown to potentially play metabolic functional roles related to disease [11]. Mok et al. (2021) [12] further reported that dysbiosis of the gut mycobiome in relation to AD, e.g., *Rhodotorula* sp. abundance, occurred using ITS2 sequencing and targeted metaproteomics. However, the protein expression and functions of the gut bacteriome and mycobiome in Thai infants with AD is poorly characterized through integrative data analysis.

This study therefore aimed to investigate the protein expression, functions and interactions of the gut bacteriome and mycobiome as well as the human proteome in Thai infants with AD using integrative metaproteomic and host interaction analysis. Briefly, metagenomic data were collected to identify the predominant species. Then, the metaproteome data of healthy infants and those having AD were compared in protein expression, functional capabilities and interactions. Additionally, the human proteomic data were also considered in relation to AD. This study highlights the potential bacterial and fungal communities and their functions as well as the interactions related to healthy and AD patients in Thai infants.

## 2. Results

### 2.1. Identifying the Predominant Taxa of the Gut Bacteriome and Mycobiome

**Figure 1:**
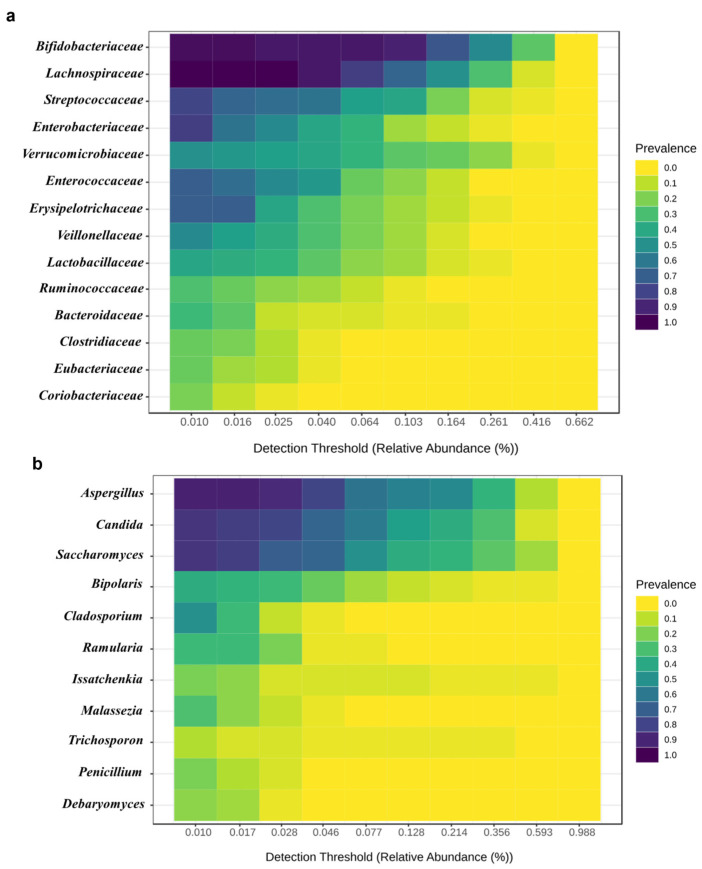
The most predominant (**a**) bacterial and (**b**) fungal taxa in Thai infant guts of healthy and AD groups. Note: Blue color indicates a greater presence of microbial taxa while yellow color indicates less presence of microbial taxa.

To identify the predominant taxa of the gut bacteriome and mycobiome, the results from metagenomic data (16S rRNA gene and ITS2 region sequence data) were analyzed for healthy and AD groups. As a result, 14 predominant bacterial families (*Bifidobacteriaceae*, *Lachnospiraceae*, *Streptococcaceae*, *Enterobacteriaceae*, *Verrucomicrobiaceae*, *Enterococcaceae*, *Erysipelotrichaceae*, *Veillonellaceae*, *Lactobacillaceae*, *Ruminococcaceae*, *Bacteroidaceae*, *Clostridiaceae*, *Eubacteriaceae* and *Coriobacteriaceaeand*) and 11 predominant fungal genera (*Aspergillus*, *Candida*, *Saccharomyces*, *Bipolaris*, *Cladosporium*, *Ramularia*, *Issatchenkia*, *Malassezia*, *Trichosporon*, *Penicillium* and *Debaryomyces*) were identified as shown in Figure 1a,b. Notably, we found that *Bifidobacteriaceae* and *Aspergillus* were the predominant taxa of the gut bacteriome and mycobiome, respectively.

### 2.2. Analyzing Taxonomy, Composition and Diversity of the Gut Bacteriome and Mycobiome Between Healthy and AD Groups

**Figure 2:**
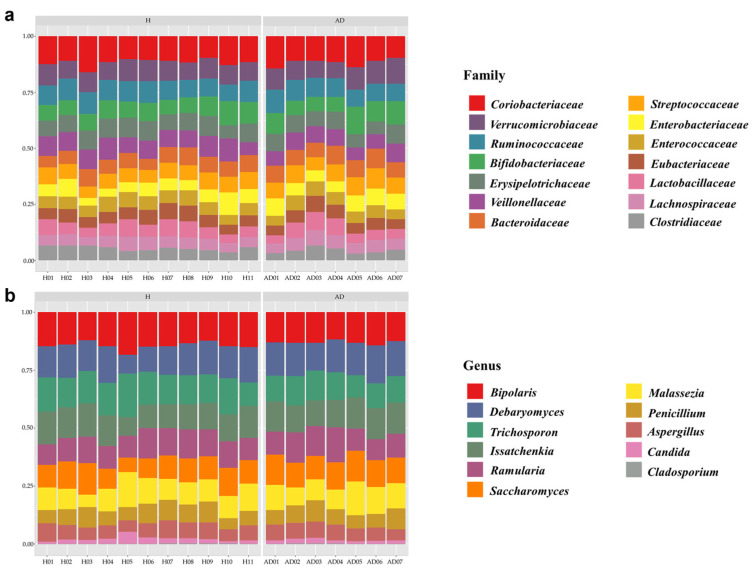
Microbial composition profiles between healthy and AD groups for (**a**) bacterial families and (**b**) fungal genera.

To analyze the taxonomy and composition of the gut bacteriome and mycobiome, the relative abundance of 14 bacterial families and 11 fungal genera were compared between healthy and AD groups, as illustrated in Figure 2. Among these bacterial families and fungal genera, we found that 1131 bacterial species and 204 fungal species were identified (Supplementary file 1). Using the Mann–Whitney U test, we found a non-significant difference in the composition of the bacterial families and fungal genera between healthy and AD groups.

**Figure 3:**
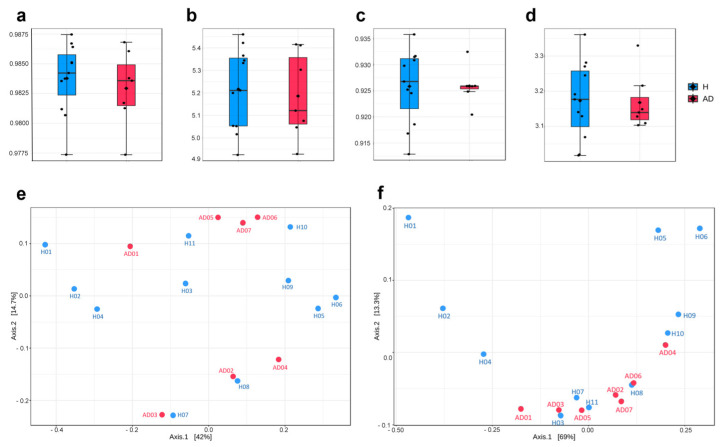
Alpha and beta diversity comparisons of gut microbiome between healthy (H) and AD groups. Boxplot shows alpha diversity indices (**a**,**b**) Shannon’s and Simpson’s for the bacteriome, respectively, and (**c**,**d**) Shannon’s and Simpson’s for the mycobiome between H (blue) and AD (red) groups. Principal coordinate analysis (PCoA) plots display beta diversity based on Bray–Curtis distance for (**e**) the bacteriome and (**f**) the mycobiome studies.

Concerning bacterial and fungal diversities, alpha and beta diversity analyses were performed for the healthy and AD groups. As a result, the alpha diversity measurements, including Shannon’s index and Simpson’s index, showed no significant differences in both bacterial and fungal diversity, as assessed using the Mann–Whitney U test (Figure 3a–d). Focusing on beta diversity, a principal coordinate analysis (PCoA) plot based on Bray–Curtis distance is depicted in Figure 3e,f. The results showed no statistically significant differences in bacterial and fungal diversity patterns between healthy and AD groups as evaluated by PERMANOVA (*p*-value > 0.05).

**Figure 4:**
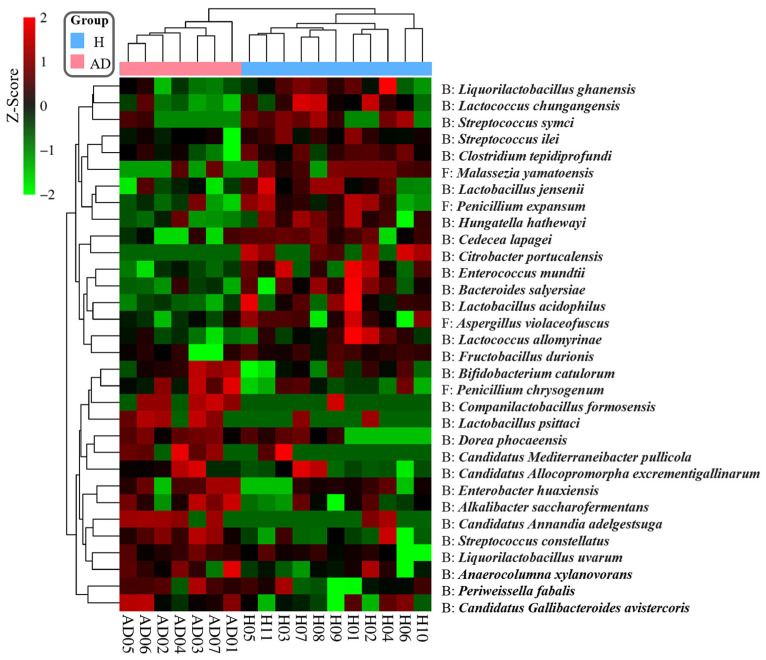
Heat map showing the relative abundance of key species associated with AD. Note: The data are log2-transformed and normalized to z-scores. Green represents reduced abundance while red indicates increased abundance. Hierarchical clustering shows a clear separation between the two groups. Each row in the heatmap represents a microbial species, classified as either bacterial (B) or fungal (F), and each column corresponds to an individual sample.

To identify key species associated with AD, the log2-transformed PELs were used to evaluate significant differences in composition using the Mann–Whitney U test (*p*-value < 0.05). Accordingly, a total of 32 potential species were identified (Figure 4 and Table 1), including 28 bacterial species and 4 fungal species. Very interestingly, we found that 14 key species (*Companilactobacillus formosensis*, *Candidatus Annandia adelgestsuga*, *Dorea phocaeensis*, *Candidatus Mediterraneibacter pullicola*, *Lactobacillus psittaci*, *Enterobacter huaxiensis*, *Liquorilactobacillus uvarum*, *Candidatus Allocopromorpha excrementigallinarum*, *Bifidobacterium catulorum*, * Periweissella fabalis*, *Candidatus Gallibacteroides avistercoris*, *Streptococcus constellatus*, *Alkalibacter saccharofermentans* and *Anaerocolumna xylanovorans*) were significantly increased in the AD group. Conversely, the other 14 key species (*Enterococcus mundtii*, *Hungatella hathewayi*, *Liquorilactobacillus ghanensis*, *Lactococcus chungangensis*, *Bacteroides salyersiae*, *Lactococcus allomyrinae*, *Lactobacillus acidophilus*, *Lactobacillus jensenii*, *Streptococcus ilei*, *Clostridium tepidiprofundi*, *Fructobacillus durionis*, *Cedecea lapagei*, *Streptococcus symci* and *Citrobacter portucalensis*) were significantly reduced in the AD group. Among the fungal species, *Penicillium chrysogenum* was significantly increased in the AD group, while three species (i.e., *Penicillium expansum*, *Aspergillus violaceofuscus* and *Malassezia yamatoensis*) were significantly reduced in the AD group. These findings show potential alterations in the gut bacteriome and mycobiome composition associated with AD, highlighting specific bacterial and fungal species that may play a role in AD development.

### 2.3. Identification of Significant Proteins and Assigned Functions Involved in AD Through DEP Analysis

**Figure 5:**
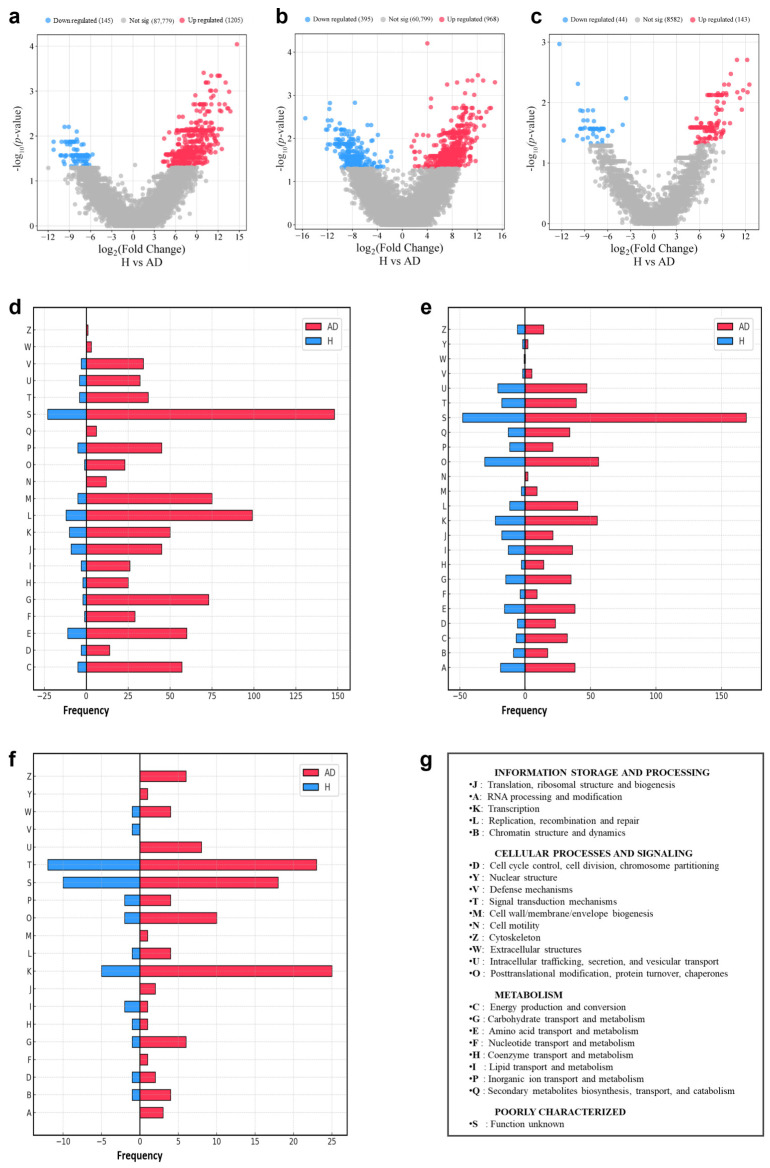
DEP analysis between healthy (H) and AD groups. Volcano plot depicts DEPs in (**a**) bacterial metaproteomics, (**b**) fungal metaproteomics and (**c**) human proteomics, respectively (red color: upregulated proteins in AD; blue color: downregulated proteins in AD; grey color: no significance). (**d**–**f**) Mirrored bar plot represents the frequency of DEPs classified by COG terms in (**d**) the bacteriome, (**e**) mycobiome and (**f**) human (red color: upregulated proteins in AD; blue color: downregulated proteins in AD). (**g**) List of COG categories and their functional terms.

To explore the significant proteins associated with AD, DEP analysis was performed for the bacteriome, mycobiome and human host. A total of 1350 significant proteins were identified in the bacteriome (Figure 5a), including 1205 upregulated proteins (89.26%) and 145 downregulated proteins (10.74%) in the AD group. For the mycobiome, 1363 significant proteins were identified (Figure 5b), comprising 968 upregulated proteins (71.02%) and 395 downregulated proteins (28.98%). For the human host, 187 significant proteins were identified (Figure 5c), including 143 upregulated proteins (76.47%) and 44 downregulated proteins (23.53%) in the AD group compared to the healthy group.

To further provide insight into functions, the annotation of significant proteins was performed. Most COG-classified significant proteins were upregulated in the AD group compared to the healthy group, particularly in the bacteriome dataset (Figure 5d). Notably, 99 out of 1205 upregulated proteins that were categorized under DNA replication, recombination and repair were majorly identified in the AD group. In the mycobiome dataset (Figure 5e), 111 out of 968 significant proteins that were associated with post-translational modification, protein turnover, chaperones and transcription were also upregulated in the AD group. Similarly, in the human host dataset (Figure 5f), 48 out of 143 significantly upregulated proteins in the AD group were classified primarily under transcription and signal transduction mechanisms.

**Figure 6:**
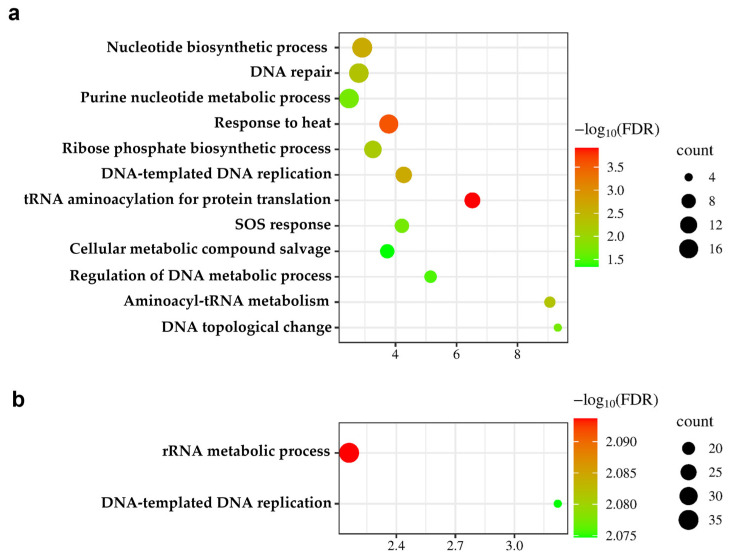
Functional enrichment analysis of upregulated proteins under the bacteriome and mycobiome datasets (FDR < 0.05). (**a**) Bubble plot shows 12 significantly enriched GO biological processes in the bacteriome. (**b**) Bubble plot shows 2 significantly enriched GO biological processes in the mycobiome. The *x*-axis represents fold enrichment while bubble color indicates -log10(FDR) and bubble size represents protein count.

Further, a functional enrichment analysis based on GO terms was performed. Interestingly, we observed upregulated proteins from both bacteriome and mycobiome datasets were significantly enriched in the GO’s biological process category (FDR < 0.05). For the bacteriome dataset, 12 significantly enriched GO terms were identified (Figure 6a), including nucleotide biosynthetic process, DNA repair, purine nucleotide metabolic process, response to heat, ribose phosphate biosynthetic process, DNA-templated DNA replication, tRNA aminoacylation for protein translation, SOS response, cellular metabolic compound salvage, regulation of DNA metabolic process, aminoacyl-tRNA metabolism involved in translational fidelity and DNA topological change. In the mycobiome dataset, two GO terms were significantly enriched in the AD group (Figure 6b): (rRNA metabolic process and DNA-templated DNA replication.

### 2.4. Revealing Keystone Functional Roles of Microbiome Through Human Host Interactions Associated with AD

To reveal keystone functional roles of the microbiome involved in AD, PPI analysis based on upregulated proteins from the bacteriome and mycobiome datasets was conducted. The PPI network using a K-means clustering algorithm of the bacteriome dataset (*Escherichia coli* K12) was explored, and it contained 91 protein nodes (Figure S1). Functional analysis of the cluster for biological process revealed several enriched GO terms, including response to stress (GO:0006950), DNA metabolic process (GO:0006259), regulation of cell shape (GO:0008360), nucleotide biosynthetic process (GO:0009165), DNA repair (GO:0006281), response to heat (GO:0009408), SOS response (GO:0009432), tRNA aminoacylation for protein translation (GO:0006418) and DNA topological change (GO:0006265). These findings show that the cluster retains the core biological functions observed in all possible upregulated proteins in the AD group. As listed in Table 2, promisingly, 13 out of 91 proteins and functional roles identified from different bacterial species were majorly involved in DNA repair, such as ribonuclease HII (rnhB), DNA polymerase IV (dinB), transcription-repair-coupling factor (mfd), holliday junction branch migration complex subunit RuvA (ruvA), UvrABC system protein C (uvrC), DNA mismatch endonuclease Vsr (vsr), DNA repair protein RecN (recN), regulatory protein RecX (recX), recombinase A (recA), DNA polymerase I (polA), DNA mismatch repair protein MutL (mutL) and DNA repair protein RadA (radA). Notably, the enrichment of DNA repair proteins in the AD group shows increased ROS levels in the gut, potentially associated with intestinal inflammation. As shown in earlier reports, it was identified that the bacterial metaproteome in colorectal cancer (CRC) and inflammatory bowel disease (IBD) patients had raised bacterial DNA repair proteins, likely due to increased reactive oxygen species in the gastrointestinal tract [13,14,15].

The PPI network of the mycobiome dataset (*Saccharomyces cerevisiae*) was explored and contained 39 protein nodes (Figure S2). As listed in Table 3, promisingly, 19 out of 39 proteins and functional roles identified from different fungal species were majorly involved in ribosome biogenesis (GO:0042254), such as serine/threonine-protein kinase TOR (TOR1), nucleolar protein 7 homolog (NOP7), 20S-pre-rRNA D-site endonuclease NOB1 (NOB1), ribosome biogenesis protein NSA1 (NSA1), U3 small nucleolar RNA-associated protein 25 (UTP25), U3 small nucleolar RNA-associated protein 10, U3 snoRNP protein (NAN1), essential nuclear protein 1 (ENP1), ATP-dependent RNA helicase (DBP7), PUM-HD domain-containing protein (UTP15), RNA helicase (MAK5), rRNA biogenesis protein RRP36 (RRP36), exosomal 3′-5′ exoribonuclease complex subunit Rrp40 (RRP40), midasin AAA ATPase 1 (MDN1), S1 motif domain-containing protein (RRP5), U3 small nucleolar RNA-associated protein 20 (UTP20), Something about silencing protein 10 (SAS10), rRNA processing-related protein (ENP2) and Large subunit ribosomal protein L3 (RPL3). Increased ribosome synthesis was observed in the fungal community associated with AD due to repair and defense mechanisms and inflammatory stress in the gut [16,17,18,19,20,21,22,23,24].

**Figure 7:**
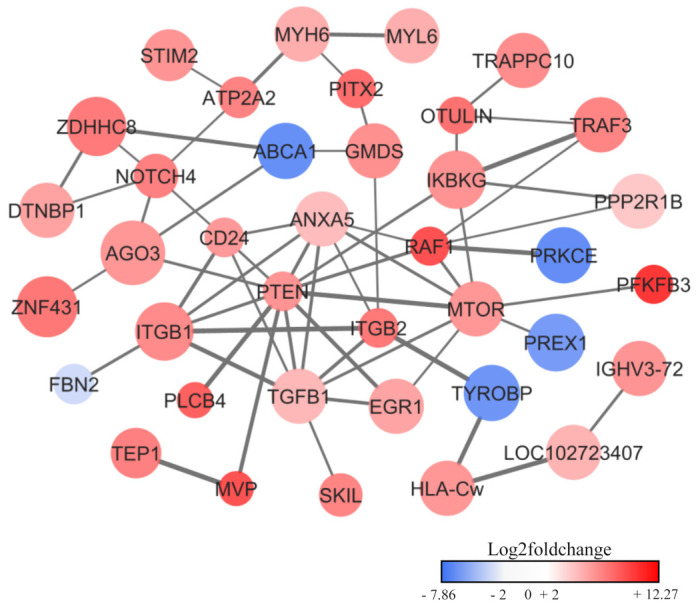
Protein–protein interactions (PPIs) network of significant proteins associated with AD. Node sizes are proportional to statistical significance, e.g., large node stands for high significance protein (low *p*-value) and small node stands for low significance protein (high *p*-value). The color of each node reflects the log2 fold change in the corresponding protein. Red node means upregulated protein and blue node means downregulated protein in AD group. The edge and edge thickness represent pairwise interactions (PIs) between proteins and strength of PIs within the network.

Beyond gut microbes, a complex interaction with the host immune system may also involve in ribosome synthesis in response to inflammation [25,26,27,28,29]. As clearly seen in the PPI network of human (*Homo sapiens*) (Figure 7)*,* 33 out of 38 proteins majorly upregulated in AD had enriched functional roles in response to stress (GO:0006950) and immune system processes (GO:0002376) (Figure S3). These findings show that stress response pathways and immune-related processes in the gut play a crucial role in the pathogenesis of AD. Interestingly, the 10 signature upregulated proteins in relation to AD had a high number of PPIs (≥5 PPIs) (Table 4). These proteins were phosphatase and tensin homolog (PTEN), serine/threonine-protein kinase mTOR (MTOR), transforming growth factor beta 1 (TGFB1), proto-oncogene c-RAF (RAF1), annexin (ANXA5), CD24 molecule (sialoglycoprotein) (CD24), Integrin beta-2 (ITGB2), Integrin beta (ITGB1), IkappaB kinase (IKBKG) and notch receptor 4 (NOTCH4). These signature proteins may play an important role in regulating gut immune homeostasis and stress responses, as well as in gut dysbiosis [15,30].

## 3. Discussion

Our detailed examination of Thai infant gut microbiomes with metaproteomics revealed differences between healthy infants and those with AD. We identified key bacterial and fungal species from feces, with 32 species showing a significant difference between the healthy and AD groups, as shown in Figure 4 and Table 1. Among these, *L. acidophilus*, a probiotic species known for its protective effects against chronic inflammation and IBD [31,32,33,34,35,36], was found to have reduced abundance in the AD group. Clinical studies have also highlighted the benefits of *L. acidophilus* in alleviating respiratory allergies [37,38]. Specifically, in the context of AD, supplementation with *L. acidophilus* L-92 in children significantly improved eczema symptoms and reduced IgE levels over a 24-week period [39]. Similarly, *B. salyersiae* was also reduced in the AD group. *B. salyersiae* CSP6 demonstrated anti-inflammatory properties in mouse models by increasing beneficial metabolites, e.g., equol, 8-deoxylactucin, and tiglic acid, while reducing *Shigella* spp. and promoting beneficial species, e.g., *Dubosiella* spp. and *Bifidobacterium pseudolongum* [40]. This reduction of *B. salyersiae* in AD patients suggested a decreased capacity for anti-inflammatory activity in the gut. Additionally, it is noteworthy that *L. jensenii* was also found to be reduced in the AD group compared to healthy infants. This bacterium has been recognized for its probiotic properties in the vaginal microbiome [41,42]. Conversely, *S. constellatus* was more abundant in the AD group. This bacterium was previously associated with significant concentrations in the mucosa and feces of patients with early-stage gastric cancer [43]. Among fungi, *P. chrysogenum* showed a significant increase in its abundance of the AD group. Previous studies have demonstrated that extracts of *P. chrysogenum* can induce stronger allergic and inflammatory responses in mice compared to house dust mite extracts [44]. Interestingly, while *M. yamatoensis* is commonly found on the skin lesions of AD patients [45], remarkably, it showed a reduction in the guts of the AD group in this study.

Moreover, our functional analysis of the DEP profiles showed a more interesting pattern. The majority of proteins were upregulated in the AD group across the bacteriome, mycobiome and human protein datasets. Notably, a COG analysis of the bacteriome highlighted proteins in the category of DNA replication, recombination and repair. A PPI network analysis of significantly upregulated proteins identified from various bacterial species showed that these PPIs were primarily associated with stress response processes, especially those related to DNA repair. These findings are consistent with the previous studies on the metaproteomes of patients with IBD and CRC, which associated these processes to elevated ROS levels in the gut [13,14]. The enrichment of DNA repair proteins in the AD group suggests increased gut ROS levels and potential intestinal inflammation [46,47,48,49]. Similarly, the PPI network of fungal species according to mycobiome dataset also showed functional enrichment in ribosome biogenesis-related processes. This likely reflects increased protein synthesis in response to gut microbiome alterations or immune system stress.

To further explore this complex interaction, we examined the human PPI network overrepresented in the AD group, which revealed a cluster of proteins associated to inflammation, immune system processes and stress response. We identified the signature proteins in the network (Figure 7 and Table 4). Among these, PTEN regulates the PI3K/Akt signaling pathway [50] and modulates immune responses in intestinal epithelial cells (IECs). A previous study showed that mice lacking PTEN in IECs had increased mortality and severe intestinal inflammation following a *Salmonella typhimurium* infection, bacterial dissemination to the organs, e.g., the liver and spleen, as well as elevated pro-inflammatory cytokine expression via NF-κB signaling [51]. PTEN also responds to DNA damage to facilitate repair [52,53]. In addition, mTOR [54] establishes a connection to inflammatory diseases such as IBD [55]. Inflammatory conditions, i.e., ulcerative colitis (UC) and Crohn’s disease (CD), demonstrate that mTOR activity in IECs is activated by microbial-derived pathogen-associated molecular patterns (PAMPs), thereby exacerbating inflammation [56]. Studies in mouse models have shown that inhibiting the PI3K/Akt/mTOR/NF-κB pathway not only reduced inflammation but also restored microbial balance by promoting the growth of short-chain fatty acid (SCFA)-producing bacteria [57,58]. TGFB1 plays a critical role in regulating immune responses and maintaining gut homeostasis. It modulates T regulatory (Treg) cells, preventing excessive immune reactions to gut microbes. Additionally, TGFB1 enhances the production of antimicrobial peptides and strengthens tight junctions in IECs, which are essential for preventing leaky gut [59,60]. In mouse models, an increase in TGFB1 expression was observed in response to radiation-induced epithelial damage, highlighting its crucial role in tissue repair [61]. ANXA5 is involved in membrane repair, which shows it anti-inflammatory properties, and it is notably abundant during IBD flare-ups [62,63,64,65,66]. ITGB2 is specifically expressed on leukocytes and plays a critical role in facilitating immune cell adhesion and migration. Its expression strongly correlates with immune cell activity (e.g., CD4+ T cells in the gut mucosa) [67,68,69]. The increased presence of this protein in the feces of the AD group may reflect the migration of these cells into the gastrointestinal lumen in response to inflammation and infection. CD24 expresses in stem cells and various immune cells which are related to regenerating tissues and immune regulation [70,71,72]. ITGB1 supports epithelial integrity and proliferation [73]. IKKB is a component of the IKK complex in the NF-κB pathway and plays a pivotal role in regulating inflammatory gene expression by facilitating the release of NF-κB into the nucleus [74]. In mouse models, the absence of IKKB in IECs results in chronic intestinal inflammation which is driven by the impaired development of T helper type 2 (TH2) cells and an overactive T helper type 1 (TH1) and TH17 response [75]. The upregulation of IKKB in the AD group may reflect an inflammatory response and/or a shift in T helper cell polarization. NOTCH4 is regarded as a regulator in inflammation which suppresses NF-κB in lung and infection models with potential parallels in gut immunity [76,77,78,79]. Finally, RAF1 is integral to the MAPK/ERK pathway implicated in cell growth and colorectal cancer. The disruption of ERK1/2 in mice led to excessive intestinal growth, but it can be restored by mTOR inhibition [80,81]. Altogether, these signature proteins illustrate the host’s coordinated response to intestinal inflammation, tissue repair and gut microbiota dysbiosis in infants with AD.

**Figure 8:**
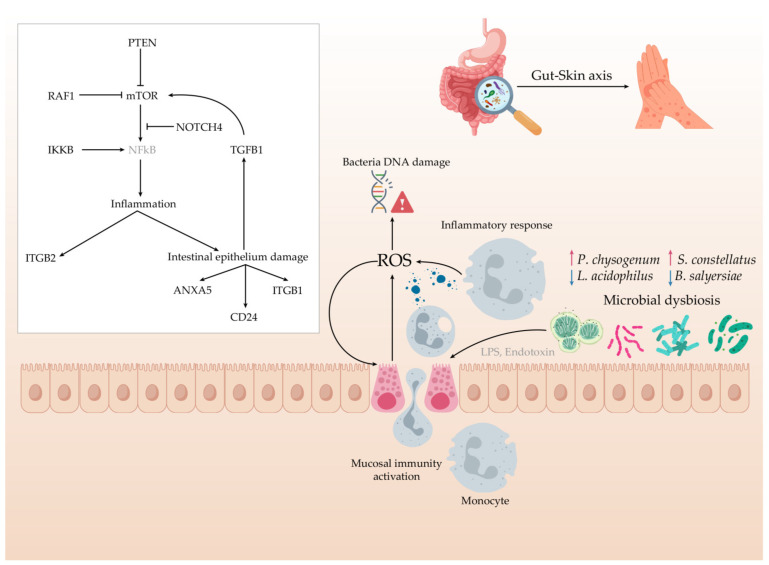
A proposed interaction of key species (arrows pointing up indicate species increased in AD, while arrows pointing down indicate species reduced in AD) and signature protein functions between gut microbes and human host in response to AD in Thai infants.

A proposed interaction of the key species and signature protein functions between gut microbes and the host in response to AD in Thai infants is illustrated in Figure 8. Our study highlights the complex interplay between the gut microbiome and the host proteome in infants with AD. The reduction in beneficial bacteria, such as *L. acidophilus* and *B. salyersiae*, underscores a diminished capacity for anti-inflammatory activity and immune modulation in the gut. Conversely, the increased abundance of *S. constellatus* and *P. chrysogenum* suggests potential roles in promoting inflammation and allergic responses. Additionally, proteins involved in DNA repair were upregulated across the bacterial population in the AD group, indicating heightened oxidative stress in the gut, likely driven by elevated levels of reactive oxygen species (ROS). This microbial stress response parallels changes in the host proteome, where upregulated proteins, such as PTEN, mTOR, IKKB and ITGB2, resulted in intestinal inflammation, reduced epithelial integrity and disrupted gut homeostasis. Collectively, these findings have shown the scaffolds of the functional consequences of gut microbiome dysbiosis and its potential linkage to host immune and inflammatory pathways, highlighting their prospective roles in the pathophysiology of AD [82].

Overall, these findings suggest several promising directions for future research into the treatment and prevention of AD by deepening our understanding of how the gut microbiome responds to AD. Further investigations with a larger cohort, including longitudinal studies and the application of additional approaches, such as cytokine profiling in the serum and skin, are required to unravel the mechanisms underlying AD development. These efforts will contribute to the development of effective strategies for preventing and treating AD and related allergic diseases.

## 4. Materials and Methods

### 4.1. Collection of Metagenomic and Metaproteomic Data

For metagenomic data collection, the pre-processed high quality 16S rRNA gene sequence and ITS2 region sequence data were provided by Patumcharoenpol et al. (2023) [10] and Mok et al. (2021) [12] for gut bacteriome and mycobiome studies, respectively. For metaproteomic data collection, raw MS/MS spectra based liquid chromatography-tandem mass spectrometry (LC-MS/MS) analyses using metaproteomic technology were collected from Kingkaw et al. (2019) [11] and Mok et al. (2021) [12] for gut bacteriome and mycobiome, as well as human proteome, studies. A total of 52 sample IDs were categorized into two groups based on the following conditions: 28 IDs of healthy infants as control group and 24 IDs of infants with AD (Supplementary file 1).

### 4.2. Identification of Predominant Taxa of Gut Bacteriome and Mycobiome Using Metagenomics

To identify predominant gut bacteriome and mycobiome taxa, 16S rRNA gene and ITS2 region sequence data were analyzed, respectively. For 16S rRNA data reported by Patumcharoenpol et al. (2023) [10], high-quality paired reads were processed using the DADA2 pipeline (v.1.10) [83] with default parameters to generate amplicon sequence variants (ASVs). Taxonomic assignment was performed with QIIME2′s naïve Bayes classifier (v.2021.8) [84] using the SILVA 99% OTU database (v.138) [85] at a 70% threshold. Microbial taxa abundance was preprocessed and filtered by the removal of ASVs with no phylum classification or relative low prevalence (<10 sample IDs). A removal of singleton ASVs was also performed to reduce a potential artifact from sequencing errors.

For ITS2 data reported by Mok et al. (2021) [12], each paired-end read was merged into a single sequence with USEARCH and subsequently filtered for quality. Merged reads shorter than 150 bps with more than 20 mismatches in alignment or an expected error >1.0 were rejected, and only the forward read was used. Operational taxonomic units (OTUs) were clustered at a 97% threshold and identified representative sequences using the UPARSE pipeline (0.8 threshold) [86]. An OTUs table was generated using USEARCH, and abundances were determined by mapping the OTUs table to the representative sequences. Taxonomic assignment was done with the SINTAX algorithm in USEARCH [87] using the UNITE fungal ITS database (v8.2) [88]. Predominant taxa were identified using the Core Microbiome function in MicrobiomeAnalyst (v2.0) [89]. The prevalence of the gut bacteriome and mycobiome was assessed using the relative abundance to calculate the percentage prevalence at the family and genus levels for bacteria and fungi, respectively. A threshold of 0.01% relative abundance and a minimum sample prevalence of 10% were applied. The potential families and genera were afterwards selected as references for further metaproteomic analysis.

### 4.3. Quantification and Identification of Microbial Proteins Using Bioinformatics Tools and Protein Databases

Protein quantification was performed using MaxQuant (version 1.6.6.0), which processed MS/MS spectra and matched them to UniProt bacterial and fungal databases via the Andromeda search engine [90]. Label-free quantification followed these parameters: a maximum of two missed cleavages, 0.6 Daltons mass tolerance, trypsin digestion, carbamidomethylation of cysteine as a fixed modification and oxidation of methionine and acetylation of the N-terminus as variable modifications. Protein databases for targeted bacterial families, fungal genera and human proteins (*Homo sapiens*) were sourced from UniProt. Protein identification was based on peptides with the highest intensity across three injections, with a minimum peptide length of 7 amino acids, at least one unique peptide and an FDR of 0.01. A maximum of five modifications per peptide was allowed, and the maximum of peptide intensities were used to determine protein expression levels (PELs).

### 4.4. Taxonomy, Diversity and Functional Analysis of Gut Bacteriome and Mycobiome

Metaproteomic data were used to assess taxonomy, diversity and functional analysis of the bacteriome and mycobiome in healthy and AD groups at 9–12 months. For taxonomy, the PELs were categorized using taxonomic information from the sequence database and then were summed to obtain total PELs for each taxonomic level (i.e., family, genus and species) in each sample ID. The relative abundance of the bacteriome and mycobiome at family and genus levels, respectively, was plotted using the bar plot in SRplot platform [91]. The Mann–Whitney U test was used to evaluate statistical differences in microbial composition at family, genus and species level between healthy and AD groups for bacteriome and mycobiome data (‘mannwhitneyu’ function, SciPy Python library, *p* < 0.05). Alpha and beta diversity analyses were performed using MicrobiomeAnalyst platform v. 2.0 [89]. For alpha diversity, Shannon’s index and Simpson’s index were used to calculate species abundance. Beta diversity was estimated using Bray–Curtis distances. Principal coordinates analysis (PCoA) was conducted to visualize the differences or similarities in microbial composition. Significant differences in microbial diversity between healthy and AD groups were identified using Mann–Whitney U test (*p*-value < 0.05) for alpha diversity and permutation analysis of variance (PERMANOVA, *p*-value < 0.05 threshold) for beta diversity.

For differential abundance of proteins, differentially expressed proteins (DEPs) analysis was performed between healthy and AD groups using total PELs. The Mann–Whitney U test (*p*-value < 0.05) and |log2foldchange| > 1 were used to identify significant proteins. For protein functional annotation of DEPs, EggNOG-mapper v2 [92] (available at http://eggnog-mapper.embl.de/, accessed on 5 September 2024) was used to assign Clusters of Orthologous Groups (COGs) terms, Gene Ontology (GO) terms and UniProt identifiers to each protein from the metaproteomic dataset. The reference orthologous groups for functional assignment of bacteriome, mycobiome and human proteins datasets were bacteria, fungi and primates, respectively, with default parameters. Additionally, functional enrichment analysis based on the GO’s biological process category was performed using PANTHER v. 19.0 [93]. Preferred names obtained from EggNOG annotation were used as input, and the analysis was conducted using Fisher’s exact test with a false discovery rate (FDR) threshold of <0.05.

### 4.5. Identification of Key Proteins Involved in AD Through Protein–Protein Interaction Analysis

**Figure 9:**
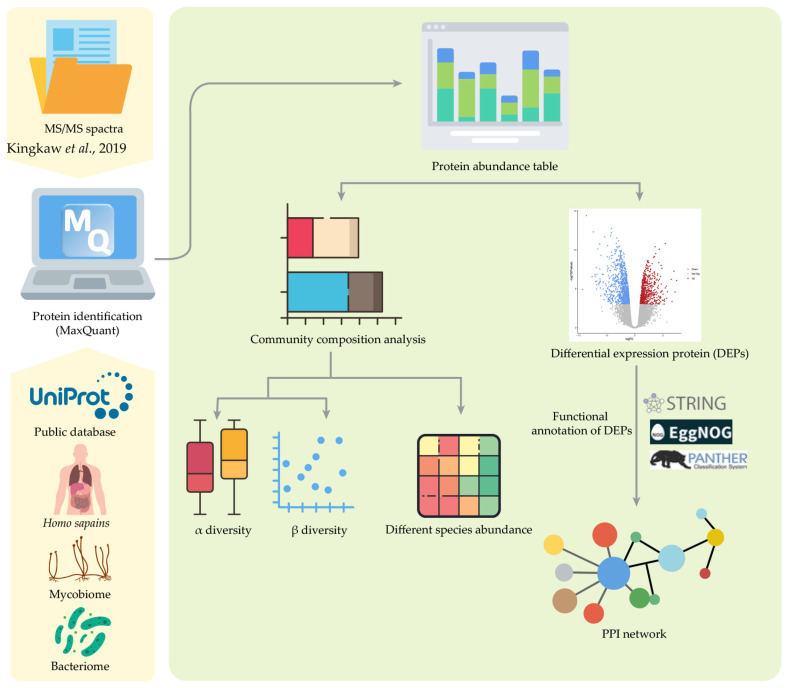
Holistic workflow of protein expression and function of the gut bacteriome and mycobiome in Thai infants associated with AD through metaproteomic and interaction analysis [11].

To identify the key proteins involved in AD, the protein–protein interactions (PPIs) for bacteriome, mycobiome and human were constructed. To explore the PPIs construction, the PPIs of *Escherichia coli* K12, *Saccharomyces cerevisiae* and *Homo sapiens* were used as templates using the STRING database v. 12.0 [94] (available at https://string-db.org/, accessed on 6 September 2024) together with the preferred names obtained from EggNOG annotation. K-means clustering algorithm was used to identify the PPI network with more than 10 nodes. To visualize the PPI network constructed for bacteriome, mycobiome and human, STRING and Cytoscape software (v. 3.10.2) was used. The holistic workflow of protein expression and function of the gut bacteriome and mycobiome in Thai infants with AD through metaproteomic and interaction analysis is presented in Figure 9.

## 5. Conclusions

Our analysis revealed the protein expression, functions and interactions of the gut bacteriome and mycobiome as well as the human proteome in Thai infants associated with AD. The key bacterial species (e.g., *L. acidophilus*, *B. salyersiae*, *S. constellatus*) and fungal species (e.g., *P. chrysogenum*) were identified towards 10 signature proteins, and related functions in stress and immune system processes in relation to the human host in Thai infants with AD were uncovered. This study serves as the first scaffold for monitoring bacteriome–mycobiome–human gut studies in infants with AD and other allergic diseases.

## Figures and Tables

**Table 1:** List of 32 potential species with increased/reduced PELs in AD.

Potential Species	*p*-Value	Log2FC	Increased (I)/Reduced (R) PELs in AD
Bacteria			
*Companilactobacillus formosensis*	0.023	7.846	I
*Candidatus Annandia adelgestsuga*	0.039	7.687	I
*Dorea phocaeensis*	0.022	7.677	I
*Candidatus Mediterraneibacter pullicola*	0.039	7.053	I
*Lactobacillus psittaci*	0.031	7.046	I
*Enterobacter huaxiensis*	0.028	6.425	I
*Liquorilactobacillus uvarum*	0.023	4.492	I
*Candidatus Allocopromorpha excrementigallinarum*	0.035	1.926	I
*Bifidobacterium catulorum*	0.044	1.804	I
*Periweissella fabalis*	0.035	1.707	I
*Candidatus Gallibacteroides avistercoris*	0.044	1.595	I
*Streptococcus constellatus*	0.008	1.545	I
*Alkalibacter saccharofermentans*	0.020	1.481	I
*Anaerocolumna xylanovorans*	0.044	0.975	I
*Enterococcus mundtii*	0.027	−0.864	R
*Hungatella hathewayi*	0.027	−1.242	R
*Liquorilactobacillus ghanensis*	0.044	−1.267	R
*Lactococcus chungangensis*	0.011	−1.531	R
*Bacteroides salyersiae*	0.020	−1.858	R
*Lactococcus allomyrinae*	0.035	−1.860	R
*Lactobacillus acidophilus*	0.006	−2.325	R
*Lactobacillus jensenii*	0.035	−2.560	R
*Streptococcus ilei*	0.027	−3.839	R
*Clostridium tepidiprofundi*	0.008	−4.902	R
*Fructobacillus durionis*	0.046	−6.050	R
*Cedecea lapagei*	0.012	−8.009	R
*Streptococcus symci*	0.033	−8.250	R
*Citrobacter portucalensis*	0.013	−10.055	R
**Fungi**			
*Penicillium chrysogenum*	0.015	1.229	I
*Penicillium expansum*	0.044	−0.629	R
*Aspergillus violaceofuscus*	0.035	−0.999	R
*Malassezia yamatoensis*	0.050	−9.104	R

**Table 2:** List of significantly upregulated proteins and functions across different bacterial species related to DNA repair associated with AD.

Preferred Name	Protein ID	Protein Name	Bacterial Species	*p*-Value	Log2FC
dinB	A0A4Q2YC86	DNA polymerase IV	*Verrucomicrobiaceae bacterium*	0.02	5.51
ligA	A0A1G5W4W1	DNA ligase	*Allisonella histaminiformans*	0.02	7.45
ligA	F4X959	DNA ligase	*Ruminococcaceae bacterium* D16	0.04	7.06
ligA	A0A1I0MYK7	DNA ligase	*Ruminococcaceae bacterium* KH2T8	0.02	6.48
ligA	R7FY99	DNA ligase	*Eubacterium* sp. CAG:841	0.02	6.17
mfd	A0A928HB07	Transcription-repair-coupling factor	*Verrucomicrobiaceae bacterium*	0.04	6.83
mutL	R5DH84	DNA mismatch repair protein MutL	*Clostridium* sp. CAG:715	0.02	6.04
polA	A0A1H3FBY6	DNA polymerase I	*Ruminococcaceae bacterium* YAD3003	0.02	7.20
polA	A0A2N3RA46	DNA polymerase I	*Bifidobacterium asteroides*	0.02	5.86
radA	A0A949U120	DNA repair protein RadA	*Clostridium thailandense*	0.03	9.27
recA	A0A087EI90	Recombinase A	*Bifidobacterium tsurumiense*	0.02	7.91
recN	A0A087CH06	DNA repair protein RecN	*Bifidobacterium psychraerophilum*	0.02	6.68
recN	A0A1H9DQR1	DNA repair protein RecN	*Lachnospiraceae bacterium* NE2001	0.02	6.50
recN	A0A413CF57	DNA repair protein RecN	*Collinsella* sp. AF11-11	0.02	6.35
recX	E6LGV7	Regulatory protein RecX	*Enterococcus italicus*	0.02	6.26
recX	A0A941HNV1	Regulatory protein RecX	*Proteiniclasticum sediminis*	0.02	5.58
rnhB	A0A2W3ZFU3	Ribonuclease HII	*Enterococcus plantarum*	0.02	6.15
ruvA	A0A430FHN6	Holliday junction branch migration complex subunit RuvA	*Bifidobacterium callimiconis*	0.03	8.10
uvrC	A0A5K1J0X0	UvrABC system protein C	*Collinsella* sp. AK_207A	0.02	5.68
vsr	A0A6L9SY74	DNA mismatch endonuclease Vsr	*Bifidobacterium platyrrhinorum*	0.02	6.69

**Table 3:** List of significantly upregulated proteins and functions across different fungal species related to ribosome biogenesis associated with AD.

Preferred Name	Protein ID	Protein Name	Fungal Species	*p*-Value	Log2FC
DBP7	A0A2N1JBU0	ATP-dependent RNA helicase	*Malassezia vespertilionis*	0.02	6.04
ENP1	A0A2N1J9C8	Essential nuclear protein 1	*Malassezia vespertilionis*	0.03	6.95
ENP2	J8QH80	rRNA processing-related protein	*Trichosporon asahii* var. asahii	0.05	5.60
MAK5	A0A1V6SJ42	RNA helicase	*Penicillium flavigenum*	<0.01	11.21
MDN1	A0A1Q5T8N0	midasin AAA ATPase 1	*Penicillium subrubescens*	0.01	7.97
NAN1	Q6BHF2	U3 snoRNP protein	*Debaryomyces hansenii*	0.04	6.49
NOB1	W7ESU5	20S-pre-rRNA D-site endonuclease NOB1	*Bipolaris victoriae*	0.02	6.22
NOP7	W7EUM3	Nucleolar protein 7 homolog	*Bipolaris victoriae*	0.02	7.16
NSA1	A0A0V1PSU5	Ribosome biogenesis protein NSA1	*Debaryomyces fabryi*	0.01	9.82
RPL3	J6EV92	Large subunit ribosomal protein L3	*Trichosporon asahii* var. asahii	0.02	7.61
RRP36	A0A135LQK1	rRNA biogenesis protein RRP36	*Penicillium patulum* (*Penicillium griseofulvum*)	0.02	7.60
RRP40	A0A2U9QXW9	K Homology domain-containing protein	*Pichia kudriavzevii* (*Issatchenkia orientalis*)	0.01	8.15
RRP40	B6GYR0	Exosomal 3’-5’ exoribonuclease complex subunit Rrp40	*Penicillium chrysogenum*	0.02	5.47
RRP5	A0A099NZW8	S1 motif domain-containing protein	*Pichia kudriavzevii* (*Issatchenkia orientalis*)	0.01	6.54
SAS10	Q12136	Something about silencing protein 10	*Saccharomyces cerevisiae*	0.04	7.84
TOR1	A0A317VPB4	Serine/threonine-protein kinase TOR	*Aspergillus heteromorphus* CBS 117.55	0.02	6.50
UTP10	Q6BXQ6	U3 small nucleolar RNA-associated protein 10	*Debaryomyces hansenii*	0.04	5.45
UTP15	A0A2N1JDU3	PUM-HD domain-containing protein	*Malassezia vespertilionis*	0.01	7.95
UTP20	P35194	U3 small nucleolar RNA-associated protein 20	*Saccharomyces cerevisiae*	0.01	6.43
UTP25	Q6BYC4	U3 small nucleolar RNA-associated protein 25	*Debaryomyces hansenii* (*Torulaspora hansenii*)	0.04	6.47

**Table 4:** List of significantly upregulated proteins and functions in humans related to stress and immune system processes associated with AD.

Preferred Name	Protein ID	Protein Name	Number of PPIs	*p*-Value	Log2FC
PTEN	A0A8V8TQX2	Phosphatase and tensin homolog	11	0.01	7.45
MTOR	A0A8V8TQM6	Serine/threonine-protein kinase (mTOR)	8	0.02	7.27
ITGB1	C9JPK5	Integrin beta	7	0.03	7.93
TGFB1	A0A7I2V5Z9	Transforming growth factor beta 1	7	0.02	5.62
RAF1	B4E1N6	Proto-oncogene c-RAF	6	0.01	10.93
ANXA5	D6RBE9	Annexin	6	0.02	5.43
ITGB2	P05107	Integrin beta-2	5	0.01	8.86
NOTCH4	A0A1U9X976	Notch receptor 4	5	0.01	8.51
IKBKG	A8K4I7	IkappaB kinase (IKKB)	5	0.03	7.53
CD24	A0A087WU21	Cluster of differentiation 24	5	0.01	7.25

## Data Availability

Data is contained within the article and Supplementary Materials.

## Supplementary Materials

The following supporting information can be downloaded at https://www.mdpi.com/article/10.3390/ijms252413533/s1.
