# Supplementary material for: Exploring Protein Functions of Gut Bacteriome and Mycobiome in Thai Infants Associated with Atopic Dermatitis Through Metaproteomic and Host Interaction Analysis
Source: Int J Mol Sci. 2024 Dec 18;25(24):13533. doi: 10.3390/ijms252413533 (PMC11676981; doi:10.3390/ijms252413533)
Supplement: Supplementary file 1 [file ijms-25-13533-s001.zip › Supplementary Figures.pdf]

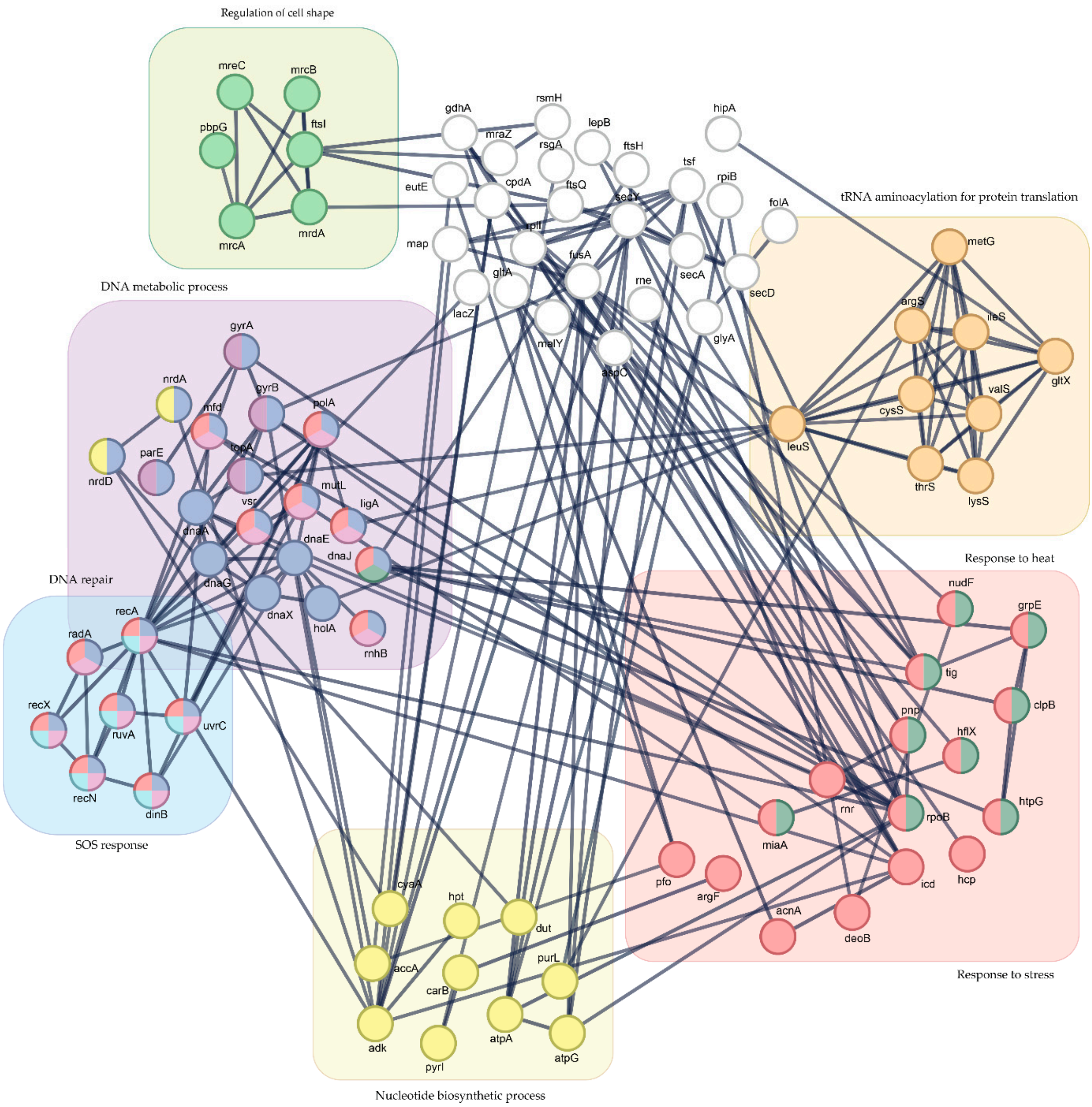

Figure S1 Main Cluster of Bacteriome PPI Network for Upregulated DEPs in AD.

This figure illustrates the primary cluster within the PPI network, highlighting 91 unique protein nodes from the bacteriome dataset that are upregulated in the AD group, as analyzed through STRING at highest confidence score. The network shows functional diversity, with several enriched Gene Ontology (GO) terms indicated by differently colored regions: Regulation of Cell Shape (green box), DNA Metabolic Process (purple box), DNA Repair and SOS Response (blue box), Nucleotide Biosynthetic Process (yellow box), Response to Stress and Response to Heat (red box), and tRNA Aminoacylation for Protein Translation (orange box). Each functional group is visually separated to enhance clarity in identifying specific biological processes linked to upregulated proteins in AD.

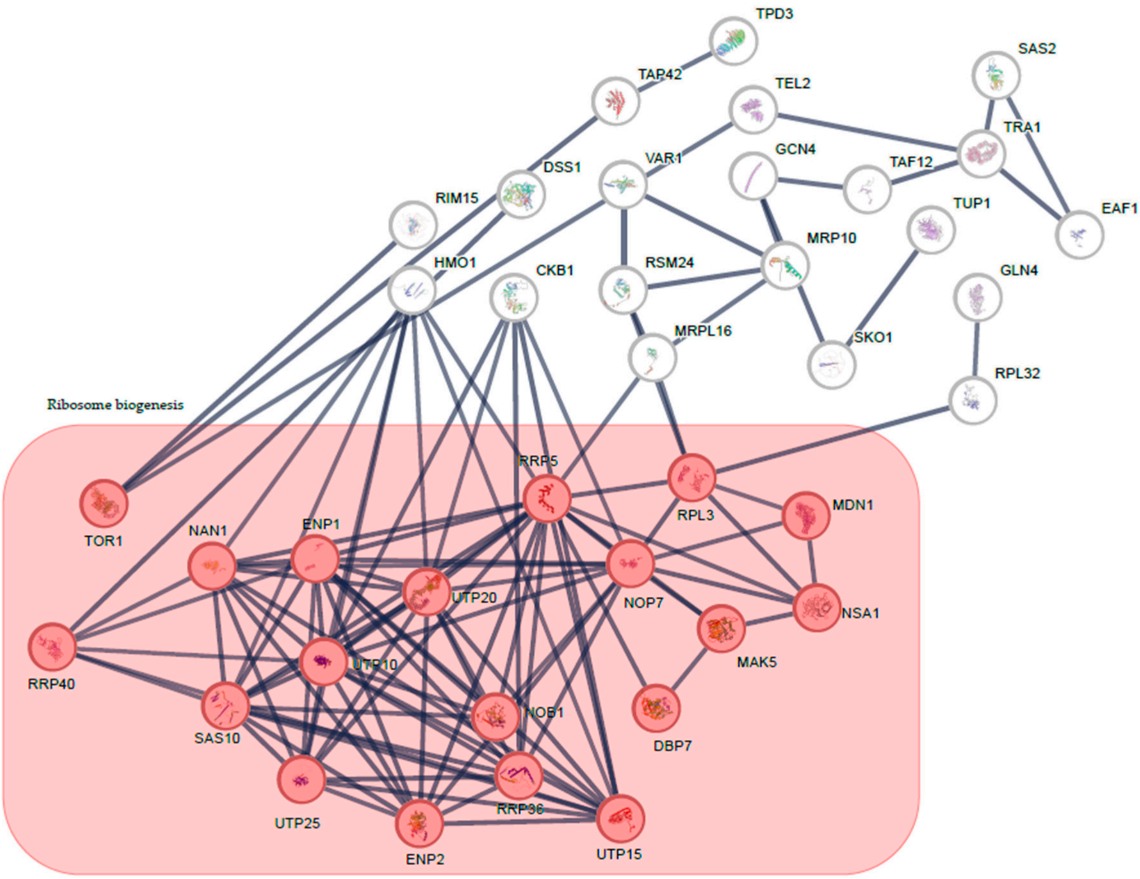

Figure S2 Main Cluster of Mycobiome PPI Network for Upregulated DEPs in AD.

This figure shows the primary cluster within the PPI network at highest confidence score, highlighting 39 unique protein nodes identified from the mycobiome dataset that are upregulated in the AD group. The primary function of this cluster is related to ribosome biogenesis (red box).

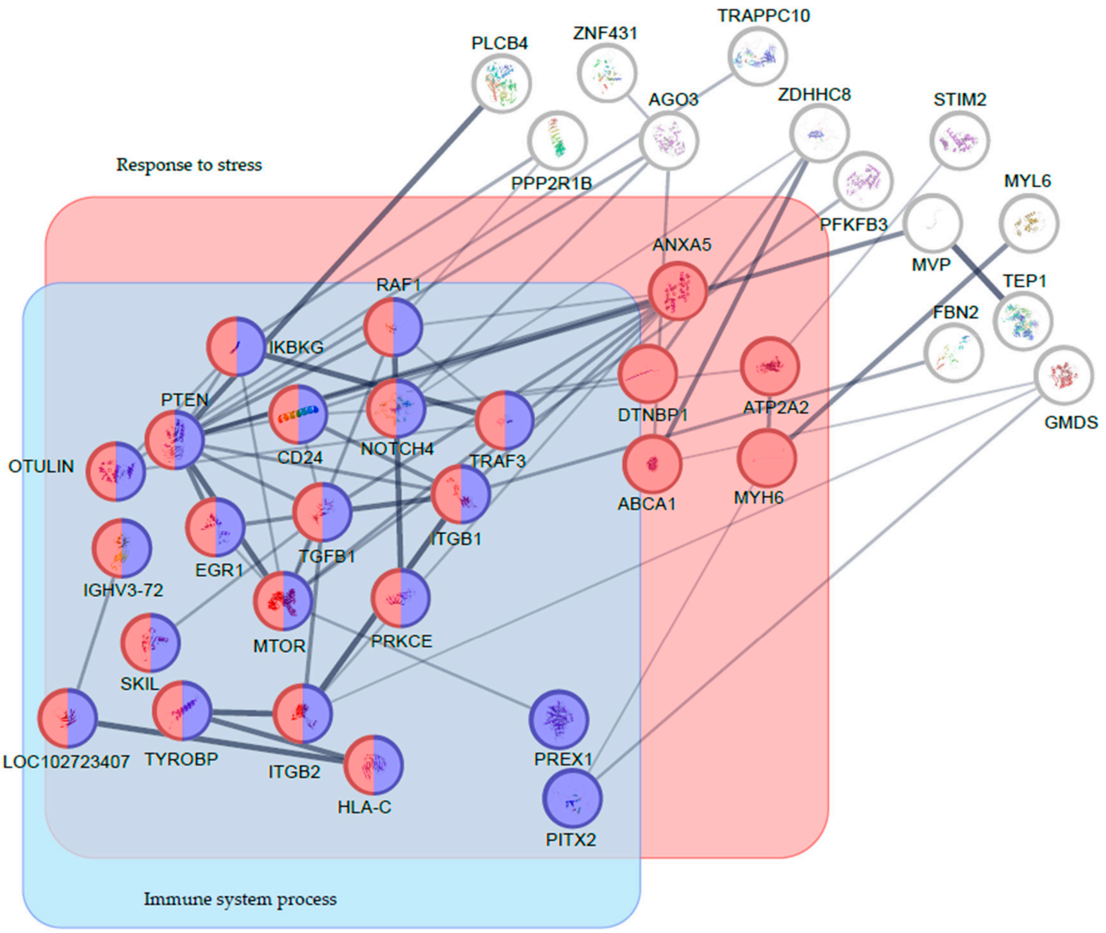

Figure S3 Main Cluster of Human PPI Network for DEPs.

This figure shows the primary cluster within the PPI network at medium confidence score, focusing on 38 unique protein nodes identified from the human dataset. Functional analysis of this cluster revealed significant Gene Ontology (GO) terms related to response to stress (red box) and immune system process (blue box).
